# Antileishmanial Activity of *Ziziphus spina-christi* Leaves Extract and Its Possible Cellular Mechanisms

**DOI:** 10.3390/microorganisms9102113

**Published:** 2021-10-07

**Authors:** Aishah E. Albalawi

**Affiliations:** Department of Biology, Faculty of Science, University of Tabuk, Tabuk 47913, Saudi Arabia; ae.Albalawi@ut.edu.sa

**Keywords:** *Leishmania major*, *Ziziphus spina-christi*, caspase 3, amastigote, BALB/c mice, Saudi Arabia

## Abstract

This experimental investigation was designed to assess the in vitro and in vivo antileishmanial effects of *Z. spina-christi* methanolic extract (ZSCME) and also aims to assess some of the antileishmanial mechanisms such as the NO production, apoptosis, and plasma membrane permeability. We assessed the in vitro leishmanicidal effects of ZSCME (10–200 µg/mL) against intracellular amastigote stage of the *Leishmania major* (MRHO/IR/75/ER) and, then, in vivo examined male BALB/c mice infected by *L. major*. In addition, the rate of infectivity, Caspase 3 activity, nitric oxide (NO) production, the plasma membrane permeability, and the cytotoxic effects of ZSCME were studied. The primary phytochemical analysis of ZSCME revealed the existence of high amounts of flavonoids, tannins, glycosides, alkaloids, and saponin in this plant. The findings exhibited that ZSCME meaningfully (*p* < 0.001) reduced the viability of amastigotes of *L. major*, whereas it prompted the creation and release of NO, apoptosis, and the plasma membrane permeability (*p* < 0.05) and indicated no cytotoxicity in macrophage cells. The in vivo results also demonstrated that ZSCME significantly decreased the parasite load and the diameter of the lesions in the infected mice. Our results demonstrate the promising in vitro and in vivo antileishmanial effects of ZSCME against of *L. major*. Although the findings of the present study showed some possible antileishmanial mechanisms of ZSCME, such as stimulating NO production, apoptosis, and increasing plasma membrane permeability, additional investigations are required to confirm these results.

## 1. Introduction

Leishmaniasis is one of the most important protozoan infections, which is triggered by the parasitic species of *Leishmania* and female sand-fly bites. About 12 million people are infected with the disease each year in 98 countries around the world, and about 350 million people are at risk for various types of *Leishmania* protozoa [1]. *Leishmania* species in humans can cause cutaneous leishmaniasis (CL), cutaneous-mucosal leishmaniasis (CML) and visceral leishmaniasis (VL) [2].

CL is one of the most important causes of chronic ulcerative skin lesions. Clinically, the disease manifests itself in many forms, including acute, chronic, recurrent and diffuse forms [3]. Cutaneous-wet leishmaniasis (rural type leishmaniasis) is one of the most prevalent forms of leishmaniasis induced by *L. major*. It is one of the zoonotic diseases (common in humans and animals) that affect many people worldwide every year [4]. In Saudi Arabia, this type of CL has the highest frequency among humans [5,6], with more than 26,000 cases having been reported in the past 10 years [7].

Various chemical drugs, such as miltefosine, pentamidine, parmomycin, allopurinol and mepacrine, are used to treat CL; however, the best choice for treatment is the use of antimony compounds such as meglumin antimoniate (Glucantim, MA) [8]. Nowadays, it has been proven that the most of the synthetic and chemical anti-leishmanial compounds have some limitations and side effects. On the other hand, some patients have reported drug resistance in treatment with MA [9].

Furthermore, treatment with these medicines is long-term, so the patient may need to use the medicine for several months [10]. The effective treatment of CL with MA usually requires injecting the drug at the site of the injury, and because the lesions are primarily visible on the hand or face of the affected person, the injection of the drug is associated with pain [11]. Due to the side effects of these drugs and chemical compounds, the use of plants and plant products native to endemic areas of the disease with minimum side effects is one of the goals of the World Health Organization (WHO) and other important global institutions [12,13].

*Ziziphus spina-christi* L. belonging to the family Rhamnaceae, which is locally called “Sidr” or “Nabuk”, grows in the wild in various different regions of Saudi Arabia [14,15]. In the past, this plant was used in Saudi traditional medicine to control palpitations, hypertension, insomnia, irritability, diabetic condition, to heal wounds, skin diseases and sores, as well as infectious diseases including ringworm infection, gonorrhea, sexually transmitted infections., etc. [16]. In modern medicine, previous studies have shown certain pharmacological properties of this plant, including antioxidant, antidiabetic, anticancer, antinociceptive, anti-inflammatory, antidiarrheic, antispasmodic and antimicrobial effects [17]. This experimental investigation was designed to assess the in vitro and in vivo antileishmanial effects of *Z. spina-christi* methanolic extract (ZSCME) and also aims to assess some of the antileishmanial mechanisms such as the nitric oxide production, apoptosis, and the plasma membrane permeability.

## 2. Materials and Methods

### 2.1. Plant Material

**Figure 1:**
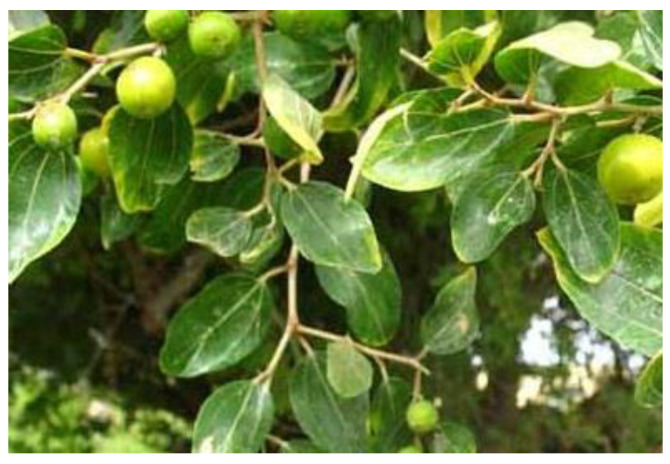
Aerial parts of *Z.* *spina-christi* used in this study.

Aerial parts of ZSC were obtained from country districts of Tabuk, Saudi Arabia (Figure 1). The collected materials were then recognized by a botanist and a sample voucher of the herb was archived at the herbarium of Department of Biology, Faculty of Science, University of Tabuk, Saudi Arabia for further experiments (No. 2020.224).

### 2.2. Preparing of Methanolic Extract

Two hundred grams of dried materials were extracted through percolation process with methanol consecutively for 3 days at 21 °C. In the next step, the extracts were passed through filter paper (Sigma, Darmstadt, Germany) and lastly evaporated in vacuum at 50 °C by means of a rotary evaporator (Heidolph, Schwabach, Germany) and kept at −20 °C until testing [18].

### 2.3. Phytochemical Analysis

In the present investigation, we performed the primary phytochemical examination of ZSCME to evaluate the presence of tannins, saponins, alkaloids, flavonoids, and glycosides, etc., based on the previous investigations [19] including: Mayer and Dragendorff’s reagents for determining the alkaloids, Mg and HCl for identification of flavonoids, 1% gelatin along with 10% NaCl solutions for determining the tannin, the combination of FeCl_2_ and H_2_SO_4_ for testing the glycosides with, and saponin with capacity to produce suds.

### 2.4. Secondary Metabolites Contents

Total phenol content was measured based on Folin–Ciocalteau’s reagent colorimetric method using gallic acid as standard [20]. In brief, 0.2 mL of ZSCME was mixed with 2 mL Folin–Ciocalteu solution and then 2 mL of sodium carbonate (7%) was added to the mixture. After 30 min of incubation in the dark, the absorbance of the suspension was measured at 750 nm by means of a spectrophotometer; whereas the total phenol content was reported as mg gallic acid equivalents (GAE)/g dry weight.

Total flavonoid content was measured by aluminum chloride (AlCl_3_ 2%) colorimetric method using quercetin as standard according to the methods elsewhere [21]. Briefly, 0.2 mL of extract or standard solution was added to 0.2 mL of aluminum chloride and 0.1 mL of 33% aqueous acetic acid and stirred well. Finally, the reaction mixture with 90% ethanol was made to a volume of 5 mL. The tubes were kept at room temperature for 30 min. Finally, the absorbance was read at 430 nm and the total flavonoid was obtained using a standard curve as mg quercetin equivalent per gram dray weight (mg QE/g DW).

The tannin condensed contents was measured according to the method described previously [22]. To achieve this, extract and control (Catechin) were mixed with 5 mL vanillin-HCl. After 200 min incubation, the absorbance was read at 500 nm, whereas the content was displayed as mg Catechin equivalent per gram dry weight (mg CE/g DW).

### 2.5. Parasite and Cell Culture

*L. major* (MRHO/IR/75/ER) and murine macrophage cells (J774-A1) were cultured at RPMI 1640 complemented with 15% heat-inactivated fetal calf serum (FCS), streptomycin (100 μg/mL), and penicillin (200 IU/mL), and Dulbecco’s modified eagle’s medium (DMEM) improved with 10% FCS at 37 °C in 5% CO_2_.

### 2.6. Anti-Intracellular Amastigote Effects

To determine the effect of ZSCME on the amastigote form, 5 × 10^5^ macrophage cells were poured in sterile 6-cell plates (with 1 cm2 cover slips implanted on their floor) and incubated at 37 °C for 24 h with 5% CO_2_ to adhere to macrophages. After 24 h, the plates were removed from the incubator and washed with sterile warm saline phosphate buffer. Then, 1 mL of RPMI1640 enriched medium containing 5 × 10^6^ *L. major* promastigotes in the stationary phase was added into plates and kept warm at 37 °C for 4 h, then the wells were washed with RPMI1640 medium to remove free promastigotes. In the next step one mL of RPMI1640 medium contains different concentrations of extract and MA were added to the wells for 48 h, the slides were then fixed with methanol and staining was then performed with Gamisa dye diluted with water in a ratio of 1:10. The results was estimated by calculating the number of amastigotes inside 100 macrophages and the number of infected macrophages in each house. The 50% inhibitory concentrations (IC_50_) were also calculated via the Probit test in SPSS software (ver. 22.0). All of the examinations in this study were carried out in triplicate [23].

### 2.7. Evaluation of the Infection Rate in Macrophages

In this study, to determine the inhibiting effect of the extract on infection of macrophages by parasites, promastigotes stages of *L. major* (1 × 10^6^/mL) were incubated in the extract (5 μg/mL) for 120 min at 21 °C. (These concentrations have no toxicity on promastigote viability according to the primary experiments). In the next phase, the promastigotes were first washed and then exposed to macrophages for four hours. Finally, the slides were permanent with methanol and then stained with Giemsa dye, then using a light microscope and computing 100 J774 cells, inhibition of infection was evaluated [24].

### 2.8. Plasma Membrane Permeability

To evaluate the permeability of the plasma membrane, promastigotes (1 × 10^6^ cells/mL) were treated with different doses of ZSCME (50–200 µg/mL), Sytox green stain was used according to the manufacturer’s protocols. The promastigotes with no drug and the promastigotes treated with 2.5% of Triton X-100 (Sigma-Aldrich, Darmstadt, Germany) were measured as the negative and positive control, respectively. The plasma membrane permeability was quantified by means of a microplate reader (BMG Labtech, Ortenberg, Germany) for 4 h [25].

### 2.9. Nitric Oxide (NO) Production

In the present investigation, to assess the effect of ZSCME on the NO release of macrophage cells we used Griess reaction for nitrites. Briefly, after 72 h exposure of the macrophage cells with extract, 0.1 mL of collected supernatants were transferred into a 96-well microplate and then 60 μL of Griess reagents A and B were put within each well. Lastly, the production of NO was determined by reading the plates at 540 nm in an ELISA reader (BioTek-ELX800, Midland, ON, Canada) [25].

### 2.10. Evaluating the Caspase-3-like Activity of Extract-Treated Promastigotes

In order to determine the Caspase-3-like activity of promastigotes treated with ZSCME, the colorimetric protease (Sigma-Aldrich, Darmstadt, Germany) method was applied based on the manufacturer recommendations. The method was conducted according to the rate of color spectrophotometric formed through the release of a molecule (pNA attached to the substrate) under the enzyme caspase-3 activity. In summary, after incubating the promastigotes 10^6^ with ZSCME for 48h, they were centrifuged at 650 rpm for 5 min at 4 °C, the cell residue was lysed, and the cell lysate was centrifuged again at 20,000 rpm for 10 min. Finally, supernatant of reaction (5 μL) was added to the 85 μL of buffer and 10 μL of caspase 3 (pNA-DEVD-Ac) solution and the mixture was incubated for 120 min at 37 °C. The caspase-3-like activity was measured by means of the light absorption at 405 nm with the ELISA reader [26].

### 2.11. Cytotoxic Effects

Toxicity effects of ZSCME against macrophage cells were calculated by exposing the macrophage cells (5 ×10^5^) to different concentrations of *Z. spina-christi* methanolic extract (0 to 500 μg/mL) for 48 h in 96-well microplates at 37 °C with 5% CO_2_. Next, viability of cells was calculated by the colorimetric MTT (3-(4,5-Dimethylthiazol-2-yl)-2,5-diphenyltetrazolium bromide) assay and the results are exhibited as the 50% cytotoxic concentrations (CC_50_ values) measured by the Probit test in SPSS software. Selectivity index (SI) was also determined as the equation of CC_50_ of macrophages/IC_50_ of *L. major* amastigotes, to calculate cytotoxicity and effects of *Z. spina-christi* methanolic extract [24].

### 2.12. In Vivo Antileishmanial Effects against Cutaneous Leishmaniasis

#### 2.12.1. Establishment of CL in BALB/c Mice

In total, 32 male BALB/c mice (weighting 20–25g) 6–8 weeks’ old were randomly divided into 4 groups (8 mice per group). This research was accomplished in agreement with the recommendations of the Guide for Care and Use of Laboratory Animals of the National Institutes of Health. Moreover, the study was approved by the ethical committee of University of Tabuk, Saudi Arabia. CL was induced in BALB/c mice through subcutaneous injection of 100 μL (2 × 10^6^ parasites/mL) of *L. major* promastigotes in stationary phase into the tail of mice by means of an insulin syringe [25]. 

#### 2.12.2. Treating Infected Mice

Approximately 6 weeks after inoculation of the parasite, when CL lesions appeared, the mice were treated as follows: (i) infected mice treated with ZSCME 100 mg/kg topically once a day for 4 weeks; (ii) infected mice treated with ZSCME 200 mg/kg topically once a day for 4 weeks; (iii) infected mice receiving the intralesional injection MA (30 mg/kg/day); (iv) infected mice receiving the normal saline. By means of Vernier caliper, the diameter of lesions in the infected mice before and after the treatment was recorded. The parasite load in the treated mice was determined through smears acquired from the lesions. In the next step, the methanol-fixed smears were stained with Giemsa and the parasite load was measured by a light microscope [25].

### 2.13. Statistical Analysis

To analyze the results, we used the SPSS statistical package, version 22.0 (SPSS, Inc.). To compare the results among tested groups, we applied the unpaired samples *t*-test and one-way analysis of variance (ANOVA), and the Dunnett’s test. *p* < 0.05 was considered statistically significant.

## 3. Results

### 3.1. Phytochemical Analysis

The results of the primary phytochemical analysis of the ZSCME exhibited the presence of high amounts of flavonoids, tannins, glycosides, alkaloids, terpenoids and a lack of saponins in this plant (Table 1).

### 3.2. Secondary Metabolites Contents

The results of the analysis of the secondary metabolites of ZSCME showed that total flavonoid, phenolic, and tannin content was 14.78 ± 0.36 (mg QE/g DW), 51.33 ± 0.41(mg GEA/g DW), and 21.6 ± 1.51 (mg CE/g DW), respectively (Table 2).

### 3.3. In Vitro Antileishmanial Effects

The obtained results revealed that ZSCME considerably (*p* < 0.001) reduced the viability of amastigotes of *L. major* in a dose-dependent manner. Based on the results, the IC_50_ value was 54.6 ± 3.15 μg/mL and 47.3 ± 2.15 μg/mL for the ZSCME extract and MA, respectively (Table 3).

### 3.4. In Vivo Antileishmanial Effects

**Figure 2:**
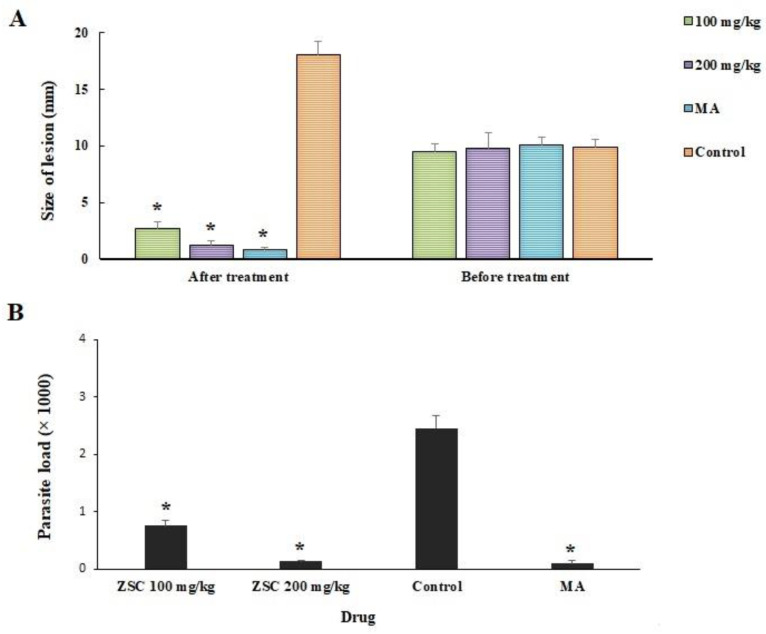
(**A**) Antileishmanial effects of *Z. spina-christi* extract on the size of lesions in BALB/c mice infected by *L. major*. * *p* < 0.001 compared with the control group; (**B**) Comparison of mean number of parasites (parasite load) in infected mice after treatment with various concentrations of *Z. spina-christi* extract compared with control group. * *p* < 0.001.

Figure 2A shows the mean size of the CL lesion in the infected mice after 4 weeks of treatment compared with the control mice. The results demonstrated that after treatment of the mice with ZSCME at the doses of 100 and 200 mg/kg as well as MA, the mean size of the lesions of was reduced by 6.4, 8.6, and 9.2 mm, respectively. However, the size of the lesions became larger by 8.2 mm in the mice treated with normal saline. Based on the obtained findings, the parasite load was meaningfully (*p* < 0.05) decreased in mice receiving ZSCME at the doses of 100 and 200 mg/kg (Figure 2B) even though the parasite load in the control group was 2.45 × 10^3^; while it was 0.75 × 10^3^ and 0.13 ×10^3^, and 0.09 ×10^3^ for ZSCME at the doses of 100, 200 mg/kg, and MA, respectively. The statistical analysis demonstrated that no significant difference was observed between in vivo antileishmanial effects of MA and ZSCME at the doses of 200 mg/kg.

### 3.5. The Effect on the Plasma Membrane Permeability

**Figure 3:**
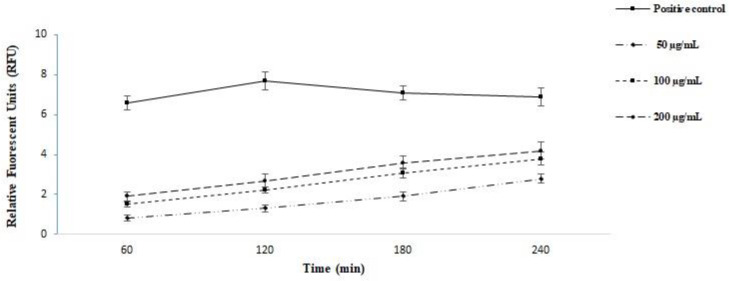
The plasma membrane permeability of the *L. major* promastigotes treated with *Z. spina-christi* methanolic extract. The findings of relative fluorescent units confirmed that the promastigotes treated with *Z. spina-christi* methanolic extract, particularly at the concentration of 200 μg/mL, change the permeability of the plasma membrane by Sytox Green.

In the present study, we assessed the plasma membrane permeability of the *L. major* promastigotes treated with ZSCME. The findings of relative fluorescent units confirmed that the promastigotes treated with ZSCME, particularly at the concentration of 200 μg/mL, changed the permeability of the plasma membrane by Sytox Green (Figure 3)

### 3.6. The Effect of Wxtract on the Infectivity Rate of Promastigotes

Based on the obtained findings, non-pre-incubated *L. major* promastigotes had the ability to infect almost 78.6% of the macrophage cells, but the pre-incubated promastigotes at the concentration of 5 µg/mL were able to infect nearly 43.6% and 30.3% of the macrophage cells (Table 4).

### 3.7. Effect on the NO Production

As shown in Table 5, *ZSCME* at the concentrations of 25 and 50 µg/mL meaningfully (*p* < 0.05) provoked the production and release of NO as a dose-dependent pattern in comparison to the non-treated macrophage cells. * *p* < 0.001.

### 3.8. Effect on the Caspase-3-like Activity

**Figure 4:**
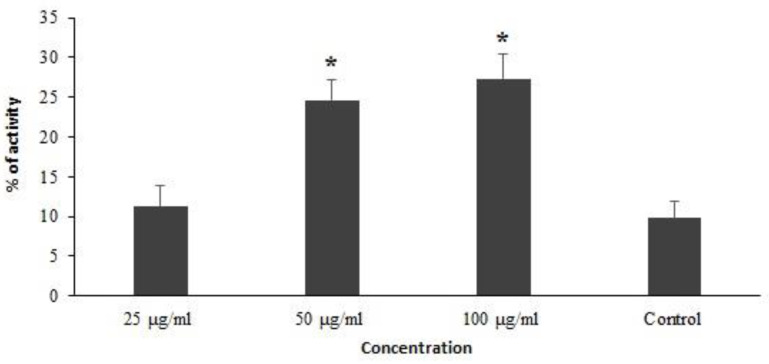
The Caspase-3-like activity of promastigotes of treated with *Z. spina-christi* methanolic extract using the colorimetric protease methods. The results demonstrate *Z. spina-christi* methanolic extract elevates the apoptosis activity in dose dependent response. * *p* < 0.05 shows the difference was statistically significant in comparison with control.

We decided to evaluate the Caspase-3-like activity of promastigotes treated with ZSCME using the colorimetric protease methods. Our findings revealed that that the ZSCME induced remarkable increased caspase-3 activation, by 11.3%, 24.6%, and 27.3%, respectively (Figure 4).

### 3.9. Cytotoxicity on the Macrophage Cells

The findings of MTT assay exhibited that ZSCME showed no significant cytotoxicity against macrophage cells. The CC_50_ value of ZSCME was 563.3 μg/mL; indicating the SI of >10 ZSCME displayed the safety of this extract for macrophage cells and specificity to the *Leishmania* (Table 1).

## 4. Discussion

Since the present treatments for LC are associated with various side effects, the use of plants and plant products native to endemic areas of the disease with minimum side effects is one of the goals of the World Health Organization (WHO) and other important global institutions [12,13]. This experimental investigation was designed to assess the in vitro and in vivo antileishmanial effects of ZSCME and also aims to assess some antileishmanial mechanisms, such as the NO production, apoptosis, and plasma membrane permeability.

The findings of the primary phytochemical analysis of the ZSCME in the present study confirmed the presence of high amounts of flavonoids, tannins, glycosides, alkaloids, and saponin in this plant. In agreement with our findings, Taghipour et al. have demonstrated the presence of flavonoids, tannins, alkaloids, and saponin in the phytochemical analysis of ethanol extracts of *Z. spina-christi* leaves [27]. Previous studies showed that these phytochemical compounds displayed significant antimicrobial effects against a wide range of microbial pathogens through some mechanisms such as changing the permeability of cell membrane, limiting the synthesis of nucleic acids and inducing cytoplasmic membrane dysfunction, disruption of energy metabolism, reduction in pathogenicity, etc. [18,19,20,21,22,23,24,25,26,27,28,29,30,31].

Our findings demonstrated that ZSCME showed potent in vitro antileishmanial effects at the IC_50_ value of 54.6 ± 3.15 μg/mL, whereas the results of the in vivo tests demonstrated that the treatment of mice with ZSCME at the doses of 100 and 200 mg/kg considerably reduced both the size of the lesions and the parasite load. 

Considering the antipaeasitic effects of *Z. spina-christi*, the results of a study by Feiz Haddad et al. demonstrate that *Z. spina-christi* hydroalcoholic extract significantly reduced the *L. major* promastigotes growth with the IC_50_ value of 112 μg/mL [32]. Another study conducted by Alzahrani et al., 2016 revealed that oral treatment of *Eimeriapap illata* infected mice with ZSCME at the doses of 100, 200 and 300 mg/kg for 5 days significantly reduced the shedding of oocysts in the feces of infected mice; in addition, it considerably improved the number of goblet cells in the jejuna villi of the mice [33]. Recently, Hafiz et al., 2019 have reported that *Z. spina-christi* leaf methanolic extract at the dose of 300 mg/kg considerably reduced the parasite load and led to a remarkable improvement of the anemic picture of hepatic injury induced in mice with *Plasmodium chabaudi* infection [34]. Almeer et al. have demonstrated that *Z. spina-christi* leaf methanolic extract at the doses of 200 and 400 mg/kg significantly improved the liver granuloma, oxidative, and fibrosis induced by *Schistosom amansoni* in CD-1 Swiss albino mice [35]. In the recent study conducted by Fadladdin et al., 2021 the obtained findings showed that *Z. spina-christi* leaf methanolic extract at the concentrations of 125, 250, and 500 μg/mL killed 100% of schistosomula and adult worms of Egyptian *Schistosoma haematobium* strains after 6–12 h of incubation [36]. 

Nowadays, it has been proven that the rupture and/or cross plasma membrane is one of the main mechanisms to inhibit the growth of intracellular microbes [28,37]. In our study, the findings of relative fluorescent units confirmed that the parasites exposed with ZSCME particularly at the concentration of 200 μg/mL changed the permeability of the plasma membrane by Sytox Green; but in positive control it permeabilized promastigotes through elevating in the detected florescence. 

Among the important factors in the pathogenicity of *Leishmania* parasites, a biological factor plays an important role in the invasion of host cells by parasites [38]. For this reason, in the present study we assessed the infectivity rate of promastigotes pre-incubated with ZSCME. Based on the obtained findings, non-pre-incubated *L. major* promastigotes had the ability to infect almost 78.6% of the macrophage cells, but the pre-incubated promastigotes at the concentration of 5 µg/mL were able to infect nearly 43.6% and 30.3% of the macrophage cells.

At present, NO, which is generated by various immune cells, plays a critical role in the immune-mediated response for eliminating intracellular pathogens such as *Leishmani* [39]. In the current work, ZSCME at the concentrations of 25 and 50 µg/mL meaningfully (*p* < 0.05) provoked the production and release of NO as a dose-dependent pattern in comparison with non-treated macrophage cells. These findings propose that while ZSCME triggered the creation of NO as a crucial intracellular antimicrobial mechanism, additional investigations and analyses nevertheless seem obligatory to assess the importance of NO and to eliminate other factors.

Apoptosis is a crucial process that naturally links an organism’s survival to its capability to prompt cell death [40]. Caspases are considered to be essential mediators of apoptosis [41]. Caspase-3 is one of the main caspases which commonly activates death protease and subsequently induces cell death [42]. Since the stimulation of apoptosis is considered to be one of the main promising antimicrobial mechanisms of tested agents, we decided to evaluate the Caspase-3-like activity of promastigotes treated with ZSCME using colorimetric protease methods. Our findings revealed that the ZSCME significantly induced the caspase-3 activation, by 11.3%, 27.3%, and 24.6%, respectively.

Regarding the cytotoxicity of the extract studied, the findings of our MTT assay showed that *Z. spina-christi* methanolic extract had an SI of >10, showing the safety of this extract for the macrophage cells and specificity to the *Leishmania* [43]. Previously, Karar et al., 2016 have demonstrated that methanolic and water extract of *Z. spina-christi* leaves had no cytotoxicity on the human epidermal keratinocyte cell line HaCa T and rat intestine epithelial IEC-6 cells in MTT assay [37].

## 5. Conclusions

Our results demonstrated the promising in vitro antileishmanial effects of *Z. spina-christi* methanolic extract against of *L. major* amastigote as well as improving the lesions of in BALB/c mice infected by *L. major*. Although, the findings of the present study showed some possible antileishmanial mechanisms of *Z. spina-christi* methanolic extract, such as prompting NO production, apoptosis, and plasma membrane permeability, additional investigations are required to confirm the antileishmanial effects and toxicity of this plant.

## Figures and Tables

**Table 1:** The primary phytochemical analysis of *Z. spina-christi* methanolic extract.

Phytochemical	Test	Presence
Alkaloids	Mayer and Dragendorff’s reagents test	+
Flavonoids	Ammonia test, alkaline reagent test	+
Glycosides	Nitroprusside test	+
Saponins	Frothing test	-
Tannins	FeCl_3_ solutions	+
Terpenoids	Salkowski test	+

**Table 2:** The results of measurement of the secondary metabolites contents of *Z. spina-christi* methanolic extract.

Total Content	Test	Amount
Phenolic	Folin–Ciocalteau’s reagent colorimetric	51.33 ± 0.41 mg GEA/g DW
Flavonoids	Aluminum chloride (AlCl_3_ 2%) colorimetric	14.78 ± 0.36 mg QE/g DW
Tannins	Vanillin-HCl colorimetric	21.6 ± 1.51 mg CE/g DW

**Table 3:** The IC_50_ and CC_50_ values (µg/mL) determined for the *Z. spina-christi* extract NPs, compared with the MA and their selectivity index (SI) against intramacrophage amastigote forms of *Leishmania major*.

Tested Material	IC_50_ (µg/mL) for *L. major* Amastigote	CC_50_ (µg/mL) of the J774-A1 Cells	SI
*Z. spina-christi* extract	54.6 ± 3.15	563.3 ± 8.63	10.31
MA	47.3 ± 2.15	914.6 ± 11.60	19.33

**Table 4:** Inhibition of the infection in macrophage cells after treatment of *L. major* promastigotes with the *Z. spina-christi* extract. Data are expressed as the mean ± SD (*n* = 3).

Promastigotes	Percentage of Infected Macrophages	Infectiveness Reduction (%)
Non-treated	78.6± 3.15	-
Treated with *Z. spina-christi* extract (5 µg/mL)	30.3± 2.51	61.4 *

* *p* < 0.05.

**Table 5:** Comparison of NO production in J774-A1 macrophage cells after treatment with various concentrations of *Z. spina-christi* extract.

Concentration (µg/mL)	Production of Nitric Oxide (nM)
10	8.6 ± 0.55
25	9.4 ± 0.64
50	16 ± 1.15 *
Non-treated	5.6 ± 1.15

* *p* < 0.001.

## Data Availability

All data generated or analyzed during this study are included in this published article.
